# Arthrogryposis Multiplex Congenita and the Importance of Orthoses: A Case Report

**DOI:** 10.7759/cureus.53993

**Published:** 2024-02-11

**Authors:** Filipa Gouveia, Luisa Pinto, Duarte Santos Sousa, João Carvalho, Catarina Aguiar Branco

**Affiliations:** 1 Physical Medicine and Rehabilitation, Unidade Local de Saúde de Entre o Douro e Vouga, Santa Maria da Feira, PRT; 2 Family Medicine, Unidade de Saúde Familiar Torrão, Administração Regional de Saúde do Norte, Agrupamento de Centros de Saúde Tâmega III, Lousada, PRT; 3 Physical Medicine and Rehabilitation, Hospital da Prelada, Porto, PRT

**Keywords:** multidisciplinary care approach, arthrogryposis multiplex congenita, clinical case report, rehabilitation outcomes, orthoses

## Abstract

Arthrogryposis multiplex congenita (AMC) is a group of conditions characterized by multiple joint contractures. This rare disorder causes stiffness of joints, limiting the range of motion and negatively impacting activities of daily living (ADL). This case reports a 45-year-old male with AMC who was referred to physical medicine and rehabilitation (PMR). He had a limited range of movement in multiple joints and global muscle weakness. However, ADL were feasible, including walking. The patient had an unsteady barefoot gait, causing claudication, which improved significantly with adapted shoes. The primary goal of treatment is to improve the quality of life (QoL), and proper management should be promptly initiated. AMC requires a multidisciplinary approach to care with three mainstays of treatment: rehabilitation, orthoses, and corrective surgeries. Patients should be followed up periodically by their family doctors, and PMR evaluations and rehabilitation should be provided as needed. An orthopedic surgery consultation may be required for surgical interventions to provide optimal outcomes and augment the QoL.

## Introduction

Arthrogryposis multiplex congenita (AMC) describes a group of heterogeneous conditions characterized by multiple non-progressive joint contractures in two or more areas, with or without muscle weakness [1-5]. Contractures vary in distribution and severity [1]. There are two major types of AMC. The most common one is amyoplasia, a sporadic form, characterized by symmetrical contractures, usually with internally rotated and adducted shoulders, overstretched elbows, flexed wrists, distal flexion contractures in interphalangeal joints, adducted thumbs, hip joint contracture (in 80% of patients), flexed or overstretched knee (in 70% of patients), and clubfoot [2-4,6,7]. The second major form is distal arthrogryposis, a group of genetic diseases with an inheritance autosomal dominant, that only affects distal parts of the limbs, hands, and feet [2,4]. The incidence of AMC ranges from 1:3000 to 1:5100 live births [4,6,8,9].

AMC causes articular stiffness, limiting the range of motion (ROM) and negatively impacting activities of daily living (ADL), such as ambulation, feeding, or toileting, and participation such as work ability, which are areas of interest in physical medicine and rehabilitation (PMR) practice [5,10-12]. AMC is usually non-progressive and often gradually improves with proper management [1]. However, no established clinical practice guidelines exist [5,10-12].

This case report aims to raise awareness of this entity in the medical community and establish the importance of multidisciplinary management and the role of rehabilitation and orthoses.

A short version of this case report was previously presented as a poster at the International Society of Physical Medicine and Rehabilitation Virtual Congress 2021 in June 2021.

## Case presentation

This case reports a 45-year-old male with AMC first seen in our PMR department as an adult. He was followed up from birth to the age of 14 years in a private hospital for multiple joint contractures, predominantly in his lower limbs. He underwent a total of nine surgeries, most of them in childhood for the management of clubfoot, the last one at 42 years of age.

Follow-up in the PMR and orthopedics departments at our hospital only started at 40 years of age, when his family doctor referred him for bilateral knee pain. He presented with a limping gait, and a radiographic assessment revealed bilateral lateral patellar deviation, high-riding patella, and external rotation of the left knee.

Two years after the initial assessment, the orthopedic surgeon performed a midfoot osteotomy with stretching of the posterior structures due to a fixed right clubfoot, which caused lower limb dysmetria. He had a posterior splint immobilization for three months, and then he progressed ambulation, first with a walker boot and then with adapted footwear. At his post-surgical PMR appointment, the patient was observed to have achieved ambulation with two crutches and refused any rehabilitation.

A year later, the patient was reportedly independent and ambulatory for short distances, requiring two crutches for longer distances. Meanwhile, he started working in a footwear factory. He made adapted shoes and insoles, improving his gait stability. He reported occasional knee and hip pain.

Two years later, a radiographic assessment showed an accentuated valgus in the left knee. Orthopaedic evaluation revealed bilateral knee instability, without progression to arthropathy; hence, he was referred to PMR.

At the PMR appointment, the patient was observed to have bilateral mechanical knee pain, worse on the left side. There was no history of chronic medication, though the patient resorted occasionally to nonsteroidal anti-inflammatory drugs for pain relief. The patient did not describe any limitations in the ADL in his married life, maintaining his job at the footwear factory, and independent ambulation with his self-made adapted footwear.

**Figure 1:**
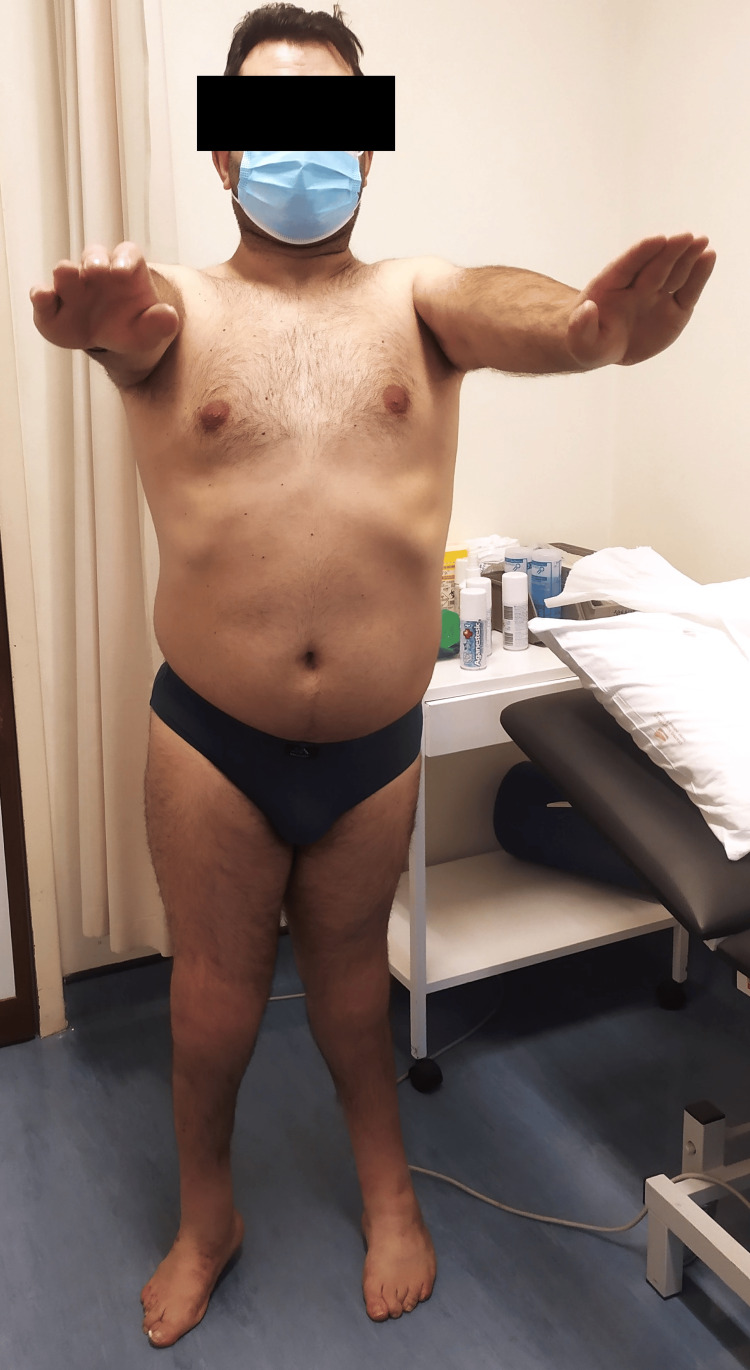
Patient's active anterior elevation of the shoulder joint, bilaterally.

**Figure 2:**
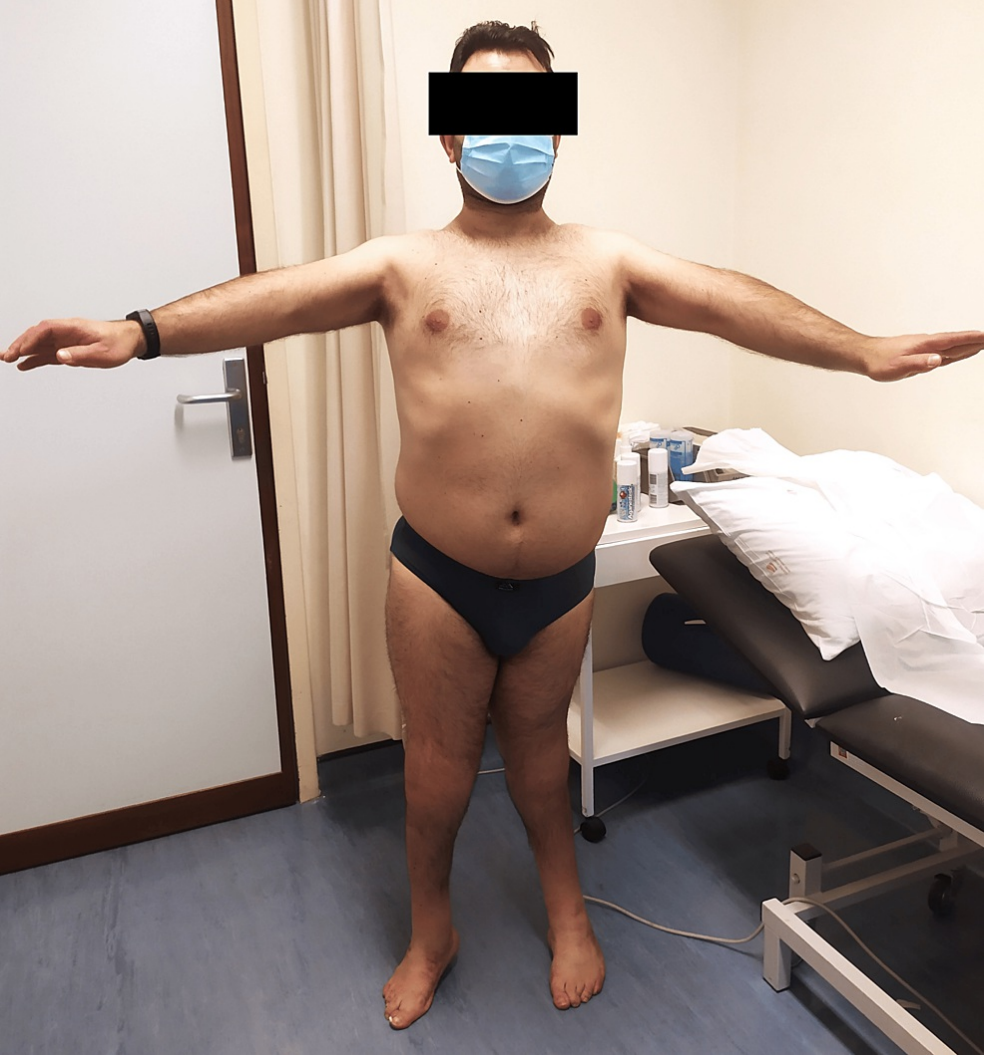
Patient's active abduction of the shoulder joint, bilaterally.

**Figure 3:**
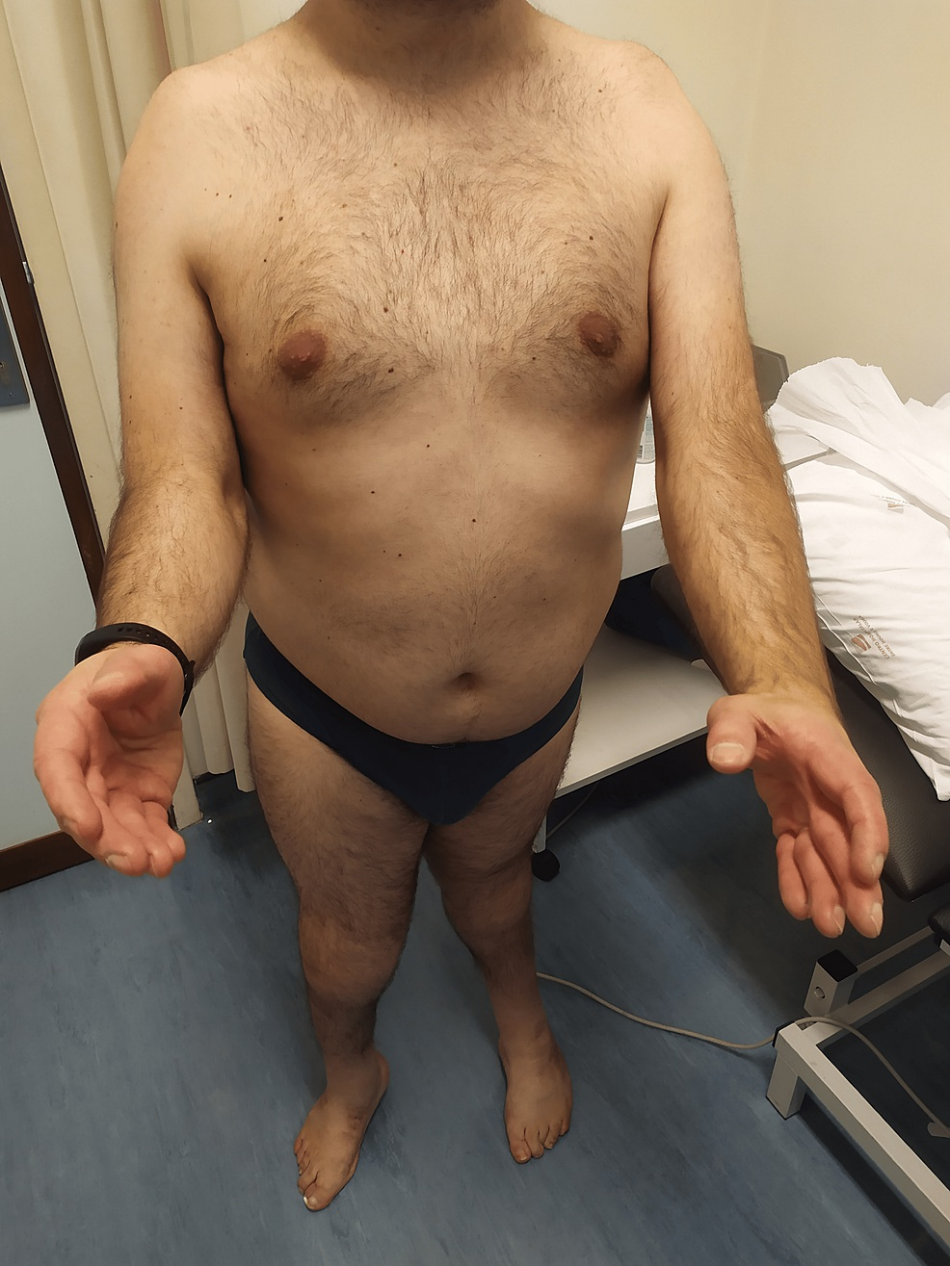
Patient's active elbow flexion, bilaterally.

**Figure 4:**
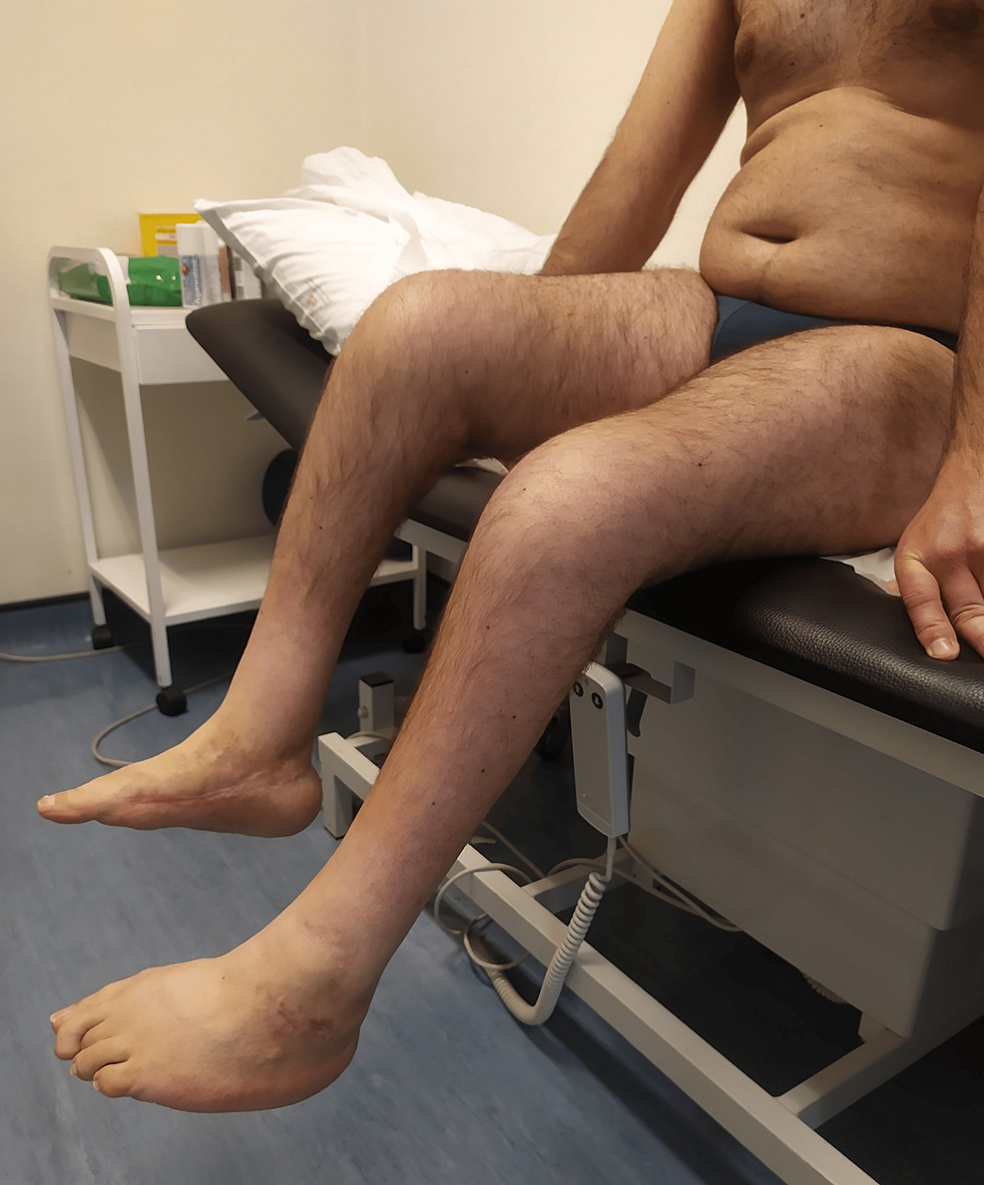
Patient's active knee flexion, bilaterally.

On clinical examination, he had ROM limitation in multiple joints, as described in Table 1. Figures 1-4 show the limited ROM in some of the joints assessed.

**Table 1:** ROM measurement of multiple joints in different planes of movement. The table specifies when the ROM is from active and passive movement and the joints with previous surgery. ROM: range of motion

Joint	Movement	ROM
Right shoulder	Anterior elevation	90º
	Lateral elevation	80º
Left shoulder	Anterior elevation	80º
	Lateral elevation	80º
Right elbow	Extension	0º (complete)
	Flexion	90º
	Pronation	80º
	Supination	60º (active), 90º (passive)
Left elbow	Extension	0º (complete)
	Flexion	60º
	Pronation	80º
	Supination	60º (active), 90º (passive)
Wrist and hand (bilateral)	No restrictions	
Hips (bilateral)	Extension	0º
	Flexion	90º
Right knee	Extension	0º (complete)
	Flexion	90º
Left knee	Extension	0º (complete)
	Flexion	70º
Right ankle	Dorsiflexion	-5º (passive; arthrodesis)
Left ankle	Dorsiflexion	0º (passive; arthrodesis)

The patient had global muscle weakness with no evident asymmetry. As per the Medical Research Council scale, he had grade 3 power in the shoulder flexors and abductors, elbow flexors, hip flexors, and knee flexors and grade 4 power in the elbow extensors, wrist extensors, palmar grip, knee extensors, ankle dorsiflexors, and plantar flexors. This assessment was carried out within the patient's limited ROM.

**Figure 5:**
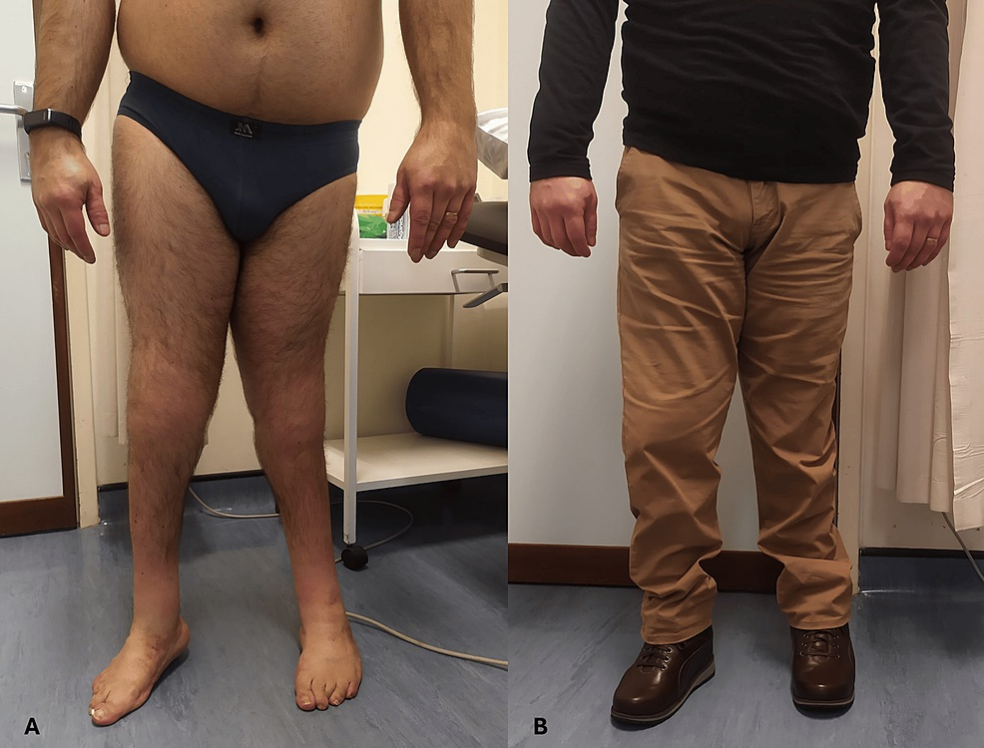
Comparison of lower limb misalignment in a barefoot condition (A) and with adapted footwear and insoles (B).

**Figure 6:**
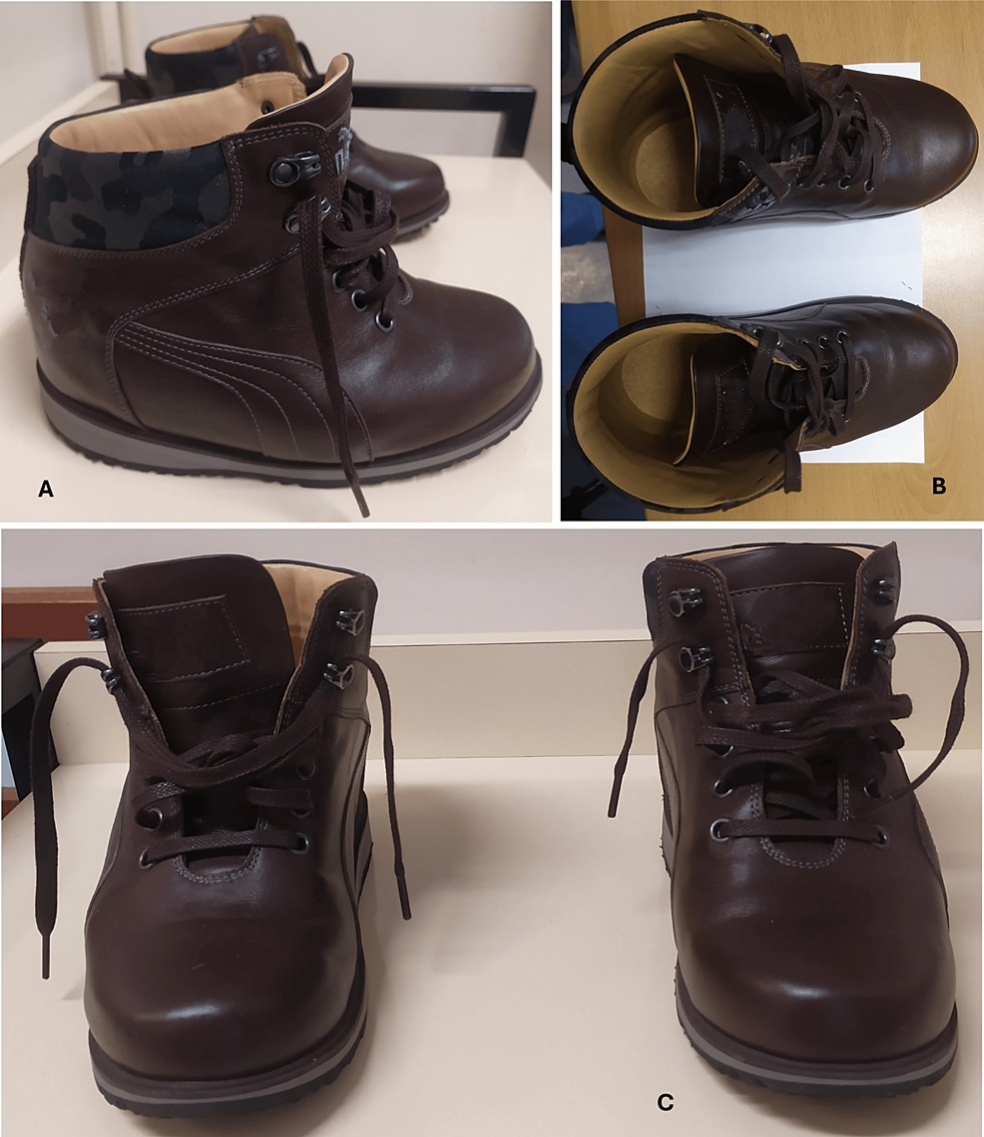
Patient's self-made adapted footwear is shown in sagittal view (A), upper view (B), and frontal view (C).

**Figure 7:**
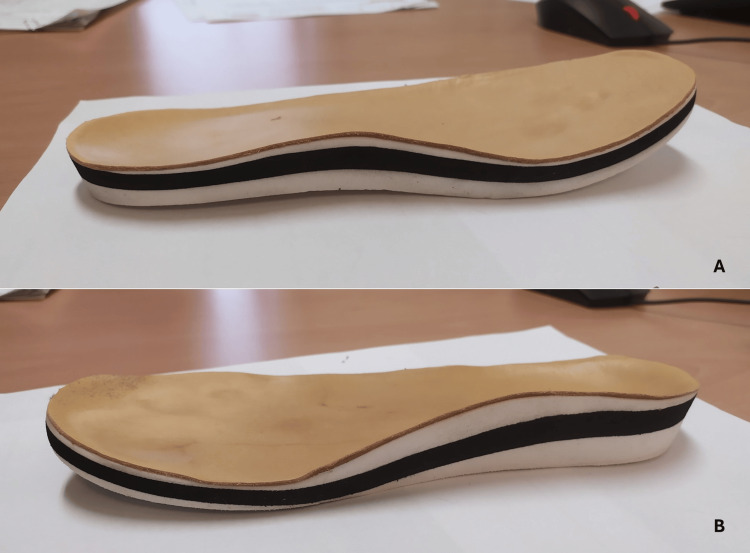
The left insole of the patient is shown above (A), and the right insole is displayed below (B).

There was a marked lower limb dysmetria and an unsteady barefoot gait, causing claudication, which improved significantly but not entirely with his adapted footwear. Figures 5A-5B show the improvement. Figures 6-7 display the self-made adapted footwear and the corresponding insoles.

A rehabilitation program was started in our department with physiotherapy and occupational therapy, including pain relievers, passive stretching, muscle strengthening, gait and balance training (including climbing stairs), ADL training, and manual dexterity exercises.

The patient understood the significance of undergoing periodic rehabilitation for his condition and expressed significant motivation to initiate and continue the treatment.

## Discussion

This report presents a rare case of AMC and its management according to current medical knowledge, although specific guidelines do not exist. The impact of AMC on mobility and ADL is variable [1]. Usually, patients have modified independence, with a mean functional independence measure score of 113 out of 126 [13].

Ambulation is the most essential goal in treating these patients. Most require human help for ADL; 50% require technical assistance, and almost 1/3 need an electric wheelchair [13]. As in this case, the literature reports that children with AMC often require multiple surgeries to achieve functional ambulation [14], with an average of 5.8 surgeries being described during a patient's lifetime [7,15]. Talipes equinovarus is the most common deformity, with a reported frequency of 98-100% of patients with AMC [6]. Clubfoot treatment aims to achieve a plantigrade foot, allowing mobilization and independent ambulation; the foot shape should accommodate orthotic devices and standard footwear [6].

On the other hand, free motion of the upper extremity joints is essential for independence in ADL (e.g. feeding, personal hygiene, and toileting), especially when muscle strength is impaired [16,17]. Therefore, muscle strengthening and manual dexterity training must be included in the periodic rehabilitation program. Additionally, assistive devices may be required. In this case, the patient was perfectly adapted to his condition, needing the foot orthoses, but with no need for any assistive device. He presented sufficient ROM and muscle strength in his upper limbs to be independent of ADL.

Chronic pain is common in patients with AMC, possibly due to the multiple surgical interventions needed and the characteristic joint misalignment [10,13].

The primary objective in treating the disease is to optimize the quality of life (QoL), focusing on unassisted ADL, empowering social engagement and independent ambulation and living [6]. Rehabilitation aims to maximize functional independence by increasing the ROM, preventing the recurrence of contractures, and addressing pain and muscle weakness [15].

This approach must be initiated as early as possible [6,18,19], including rehabilitation, individually tailored orthoses, and a broad spectrum of surgical techniques [6,15].

The rehabilitation programs should involve physiotherapy, manipulation of contractures through casting and splinting, and occupational therapy [6,15]. The most common rehabilitation techniques on AMC are stretching of the affected joints combined with splints to maintain optimum joint position and avoid worsening of contractures, passive and active joint mobilization to maintain the maximum ROM possible, and muscle strengthening to promote active muscle use [1,2,10,20]. Additionally, gait and balance training, focused on endurance and prevention of falls, as well as ADL training and manual dexterity training, are essential for promoting ambulation and functional independence, respectively [15,20]. The rehabilitation program also includes the adaptation of assistive devices that may be needed and the facilitation of activities of interest, such as computer skills or driving [17,20]. Additionally, in the individualized rehabilitation program, techniques addressing pain that is frequently present should be added [15], with the use of physical agents or massage.

Orthoses are used to maintain or correct joint mobility and prevent recurrent deformities. They may include knee-ankle-foot orthoses, ankle-foot orthoses, or adapted shoes or insoles [9,21], as in this patient.

In many cases, rehabilitation can replace invasive corrective surgery [15,19,21]; nonetheless, corrective surgery for the deformities, if needed, should not be postponed [6,15]. Orthopedic treatment, when required, should always be preceded and followed by rehabilitation [19].

The treatment of AMC is challenging due to the nature of the disease, the resulting technical surgical difficulties, and the required logistics of a complex multidisciplinary treatment [6]. The multiple joint involvements, the extensive orthopedic management, and the need for aids in ADL emphasize the importance of early referral to PMR and orthopedics [12]. The frequency of rehabilitation treatments diminishes over time, particularly in adolescence, due to the beginning of participation in leisure activities, sports, and other interests such as driving and interpersonal relations [5]. However, these patients should be followed up after adolescence [5] and re-referred as needed, highlighting the role of the family doctor.

## Conclusions

Accurate diagnosis and early initiation of AMC treatment, employing a multidisciplinary approach focused on the patient's functional independence and ambulation, are vital. The three cornerstones of AMC treatment are rehabilitation, orthoses, and surgery, with the primary goals being ambulation capacity and ADL independence, resulting in the best possible QoL. Early childhood management of AMC is essential. However, despite the non-progressive nature of this condition, adults with AMC should be routinely followed throughout their lifespan. They may experience pain and require assistive aids or rehabilitation programs to manage complaints and ensure optimal function and muscle strength.

This case report underscores the significance of physicians' awareness of this rare condition. Early diagnosis and management by a multidisciplinary team, coupled with long-term follow-up, are vital factors in ensuring optimal patient care.
